# Supplementary material for: Are carbon emissions trading and green financial instruments synergistic? -Comprehensive quantitative research based on content analysis
Source: PLoS One. 2024 Mar 7;19(3):e0298601. doi: 10.1371/journal.pone.0298601 (PMC10919601; doi:10.1371/journal.pone.0298601)
Supplement: S1 Data — (ZIP) [file pone.0298601.s001.zip › Data/地级市Z_F工作报告.zip!/╔┬╬≈/╬≈░▓╩╨/└┤╫╘╣1⁄2╓┌║┼í╛┬φ┐╦╩2╛▌═°í┐_2018.docx]

各位代表：

现在，我代表市人民政府向大会报告工作，请予审议，并请市政协委员及其他列席人员提出意见。

一、2017年政府工作回顾

过去的一年，是大西安追赶超越进程中极为重要的一年。一年来，在省委、省政府和市委的坚强领导下，我们认真学习贯彻习近平新时代中国特色社会主义思想，对标“追赶超越”定位和“五个扎实”要求，紧扣“五新”战略任务[1]和“聚焦三六九，振兴大西安”[2]奋斗目标，全面推进“九大关键行动”[3]，较好完成了市十六届人大一次会议确定的各项目标任务，大西安建设实现精彩开局。

——扎实推进经济持续健康发展，多项指标创近年新高。生产总值完成7469.85亿元，同比增长7.7%（原口径增长8.5%），总量在15个副省级城市中进至第9位，实现历史性跨越。签约引进项目847个，投资规模达2.35万亿元，为历年最高，被环球网评为“2017最受国际关注中国投资城市”。全社会固定资产投资增长12.9%，同比提高10.9个百分点。社会消费品零售总额4329.5亿元、增长10.5%。进出口总值2545.4亿元、增长39.1%，增速位居副省级城市第二。一般公共预算收入完成654.5亿元、增长9.7%，其中税收增长20.4%，位居副省级城市第一。市场主体总数达到101.6万户，成为全国第7个过百万的副省级城市。净增“五上”企业[4]685户，创近五年最高。科技进步对经济增长贡献率达到60%。

——扎实推进农业现代化建设，特色农业发展亮点突出。都市型现代农业“一区三带七板块”[5]格局不断完善，苗木花卉产业基地面积达到73.4万亩，临潼石榴获国家地理标志保护产品认证，蓝田大杏种植系统入选中国重要农业文化遗产。农产品检测合格率稳定在97%以上。新增市级以上现代农业园区20个、产业化龙头企业27家。休闲农业接待游客2350万人次、实现综合收入24.1亿元。蓝田全域旅游经验在全国推广。泾河新城茯茶镇获批中国乡村旅游创客示范基地。全市粮食生产实现“十四连丰”。

——扎实加强文化建设，文化软实力显著增强。《西安市不可移动文物保护条例》正式实施，文物保护立法走在全国前列。新增“西安城市记忆”等5座博物馆，总数达到126座。汉长安城未央宫遗址入选国家考古遗址公园名录。新增规模以上文化企业64家，文化产业营业收入增长25.2%。话剧《麻醉师》荣获全国“五个一”工程优秀作品奖，《大秦帝国之崛起》《白鹿原》《那年花开月正圆》等一批影视佳作全国热播。“西迁精神”薪火相传，“烟头不落地”“车让人”行动掀起城市文明新风尚，成功蝉联“全国文明城市”。举办第2届中法文化论坛“西安日”、第16届西安国际音乐节、2017西安电竞产业峰会、第2届全国道教文化艺术周等重大活动，西安的文化影响力明显增强。

——扎实做好保障和改善民生工作，群众有了更多获得感和幸福感。着力破解民生“九难”[6]，129项民生重点提升工作和“十个一”民生工程[7]顺利推进。城乡居民人均可支配收入达到38536元和16522元，分别增长8.2%和8.8%。全面打好脱贫攻坚战，5.4万贫困人口实现精准覆盖。打通断头路27条，新购1900辆纯电动公交车，有效缓解了市民“出行难”。改造城中村（棚户区）19个，回迁安置群众2.63万人，改造老旧小区269万平方米，18万群众居住条件明显改善。下大气力解决长期困扰市民的房产办证遗留问题，为5.5万户群众消除了“烦心事”。新建改造厕所1668座，获评“全国厕所革命优秀城市”。全面实行小学低年级“弹性离校”制度，10万余家庭从中受益。为65岁以上老年人实施增项免费体检，惠及群众62万人。改造提升农贸市场25个、规范整治111个。全力保障公众“舌尖上的安全”，成为全国首批“国家食品安全示范城市”。连续6年荣获“中国最具幸福感城市”称号。

——扎实落实全面从严治党，作风建设取得突破性成效。严格落实中央八项规定精神和“三严三实”要求，推进“两学一做”学习教育常态化制度化，用好用活“三项机制”，大力营造干事创业、比拼争先的浓厚氛围。建立了月擂台、季点评、常态化互学互比的工作机制，打造了《电视问政》《每日聚焦》《党风政风热线》等一批强化监督、治庸治懒的有效平台，“用心工作”、当好“五星级店小二”已成为全市干部的思想共识和行动自觉。我市干部作风在省上组织的群众满意度调查中创8年来最好成绩。

一年来，我们主动对标中省部署要求，积极践行新发展理念，始终坚持以人民为中心的发展思想，担当尽责、埋头苦干、兑现承诺，主要做了九方面工作：

（一）全力开启大西安建设新格局。西咸一体化步伐明显加快，与咸阳签订战略合作框架协议，全方位对接机制日臻完善，“八个一体” [8]深入推进。出台支持西咸新区加快发展系列政策，完成了环保、国土、交通等6项规划的对接融合，户籍同城、社保同城和车牌照同号工作全面落实。站高谋远做实，确立了大西安“多轴线、多中心、多组团”的空间格局和“北跨、南控、西进、东拓、中优”的发展思路。大西安综合交通规划修编完成，西成客专开通运营，西安至韩城等4条城际铁路[9]和外环高速（南段）等4条重点公路[10]开工建设。加快推进富阎一体化，产业合作园区规划编制完成，富阎连接主线实现贯通。全市人民期盼已久、对西安未来发展至关重要的《关中平原城市群发展规划》获国务院批复，明确了西安建设国家中心城市的目标定位，为奔跑的大西安插上了腾飞的翅膀。

（二）着力打造转型发展新优势。坚持以供给侧结构性改革为主线，全力补短板、调结构、促转型、增效益。三次产业结构调整为3.8∶34.7∶61.5，服务业对经济增长的贡献率达到70.4%。非公经济占比达到53%。民间投资3120亿元、增长11.1%，同比提高17.2个百分点。城镇化率达到73.4%。万元生产总值能耗下降4.6%。

叫响做实工业强市战略。出台强工业系列政策措施，六大千亿级产业集群[11]加速壮大，汽车产业迈入千亿级。新增法士特、乐叶光伏2户百亿级工业企业，总数达到11家。规模以上先进制造业总产值3167.7亿元、增长20.6%。工业企业技改投资238.8亿元、增长43.2%。创建“中国制造2025”试点示范城市通过国家评估。获批国家通用航空产业综合示范区。

现代服务业成为拉动经济增长的主力军。丝路国际金融中心建设步伐加快，新增新三板挂牌企业26家，金融机构存款余额突破2万亿元。京东、海航、传化等一批重大现代物流项目相继落地，物流业增加值728亿元、增长14.7%。举办2017西安国际通航大会等规模以上会展活动199场，会展业综合收益达到115.4亿元，创历史新高。新增3A级以上景区8个。接待海内外游客1.8亿人次、增长20.5%，旅游业总收入1633.3亿元、增长34.6%。科技、体育、康养、电竞等新兴服务业迅猛发展。

积极打造特色经济发展平台。首批35个特色小镇建设全面启动，灞柳基金等12个特色小镇投入运营。数字经济、共享经济、体验经济等新经济蓬勃发展。

（三）强力引资引智聚集新动能。把招商引资作为“一号工程”，坚持“五资”[12]齐抓，组建市投资委，成立24个专业招商机构，出台精准招商系列政策措施，为企业量身定制全要素、全周期、优质化服务。加强城市营销，充分发挥驻外机构、贸促会以及商会、校友会等社会组织作用，赴外精准招商3000余批次，接待国内外客商210批次，成功举办首届世界西商大会、全球硬科技创新大会、全球程序员节、丝绸之路工商领导人峰会等重大节会活动，在全球化的舞台上奏响了“西安乐章”。三星二期、吉利、开沃、华润、华侨城、阿里巴巴、腾讯、亚马逊等一大批重大项目签约落户，涉及世界500强企业44家，新增投资过百亿元项目40个。全年实际引进内资2186亿元、实际利用外资53.1亿美元，分别增长34.1%和17.8%。实施户籍、人才和创新创业“三大新政”，全年新增落户人口25.7万人，人才净流入量居全国大城市前列。建成高新咖啡街区、经开创业大街、曲江创客大街等众创空间422家、面积1389万平方米，累计入孵企业11529家，吸引创业就业人数22.9万人。建成西部首个“国字号”人力资源服务产业园。中科院西安光机所、西安电子科技大学获批国家“双创”示范基地。持续增强的“西引力”，为大西安加速奔跑汇聚了“排浪式”的强大势能。

（四）全面创新改革增添新活力。总结提炼24条创改经验上报国家，居全国8个试验区之首。高新自创区建设取得新进展，国家技术转移西北中心特色平台建成运营。统筹科技资源改革不断深化，技术合同交易额809亿元，位居副省级城市第一，获批“国家知识产权运营试点城市”和“国家知识产权强市创建市”。出台支持军民融合产业发展7项政策，民参军企业达到400家，军民融合产业营业收入突破2000亿元。“放管服”改革持续深入，规范整治市场秩序扎实有效，全年新登记各类市场主体28.3万户，是上年的两倍。推进国有企业股权多元化改革，实施了13个重大资产重组项目。落实“房子是用来住的，不是用来炒的”要求，及时出台控房价、防风险、稳预期调控政策，商品房去化周期缩短为10个月。通过清理涉企收费、减免政府性基金、提供税收优惠等措施，为企业降成本95.9亿元。企业家信心指数和企业景气指数持续攀升。在全省统计调查中，企业家对我市投资环境总体满意度达98.3%。

（五）加速驶入双向开放快车道。深度融入“一带一路”建设，与丝路沿线国家和城市的联系更加紧密。西安综合保税区二期通过国家验收，西安航空基地综合保税区正式获批，新增整车进口等3个口岸。开通全国首趟北欧（芬兰科沃拉—西安）国际货运班列，“长安号”开行480列，运送货物65.9万吨。新增国际客运航线14条、货运航线3条，机场旅客吞吐量4187万人次，位列全国第八。跨境电商公共服务平台上线运营。自贸试验区建设加快推进，企业注册登记实现19项事项联办和全程电子化，新增企业8898家，其中外商投资企业102家。中国贸促会陕西自贸区服务中心、丝绸之路仲裁中心、丝绸之路国际总商会落户西安。开播全国首家丝路电视频道，成功举办2017欧亚经济论坛、丝绸之路经济带城市圆桌会议等活动，国际友城达到31个，21家外事机构签约入驻。“走出去”力度不断加大，陕鼓、陕汽、西电、爱菊、隆基股份、华山国际等一批重点企业海外知名度日益提升。开放的西安正以崭新的姿态阔步迈向世界！

（六）聚焦“四治一增”[13]优环境。积极落实铁腕治霾“1+1+9”行动方案[14]，用有效的措施、不懈的坚持和铁一般的担当，努力消除百姓的“心肺之患”。全面实施“煤改洁”，拆除燃煤锅炉994台、小燃煤设施929台，削减散煤233.4万吨。清理整顿“散乱污”企业1531户，45家重点污染源单位向社会公开实时监测数据。对全市工地实行“红黄绿”挂牌管理，三环内“两类企业”[15]全部搬迁。淘汰黄标车1.48万辆，实施冬防期机动车常态化限行。全年二氧化硫、氮氧化物、烟尘排放分别减少1.2、0.7和1.4万吨，空气质量明显好转。统筹推进“五策治水”[16]和“八水绕长安”生态恢复工程，在全省率先实施“河长制”“湖长制”，渭河、灞河等9个生态示范河（湖）建设[17]有力推进，昆明池七夕公园、渼陂湖一期等建成开放，涝渭湿地、天桥1号湖实现蓄水，唐家寨陂塘治理工程全面完成，新建、改扩建污水处理厂4座，建成生活污水治理示范村50个，新增生态水面3088亩、湿地6017亩，顺利通过国家水生态文明城市建设试点验收，水韵西安的生态画卷正在向世人展开。严格落实秦岭生态环境《保护条例》和《管理办法》，推进“多规合一”，建立“四级网格”管理体系，“四乱现象”[18]得到有效整治。秦岭国家植物园建成开放。大力开展烟头革命、厕所革命和城乡环境大整治行动，全面推行“路长制”“所长制”，强力推进“四改两拆” [19]，全年治理拆除违法建设1248万平方米，拆除广告牌匾1.8万处，建成亮化示范街40条，亮化楼宇1369座。干净、整洁、优美的城市环境，赢得了市民和游客的“好口碑”。深入推进“美丽西安·绿色家园”行动，大力开展“五路”两侧增绿美化[20]，启动建设西安城市生态公园和城市中央公园，建成曲江文化运动公园等3个主题公园和62个绿地广场、86条绿化示范路，新增城市绿地571万平方米、造林绿化4.27万亩。广大市民的“幸福账单”上，又增加了不少看得见、摸得着、感受得到的“绿色收入”。

（七）持续增强城市功能提品质。积极推进“强基增容”工程，完成城建投资677.3亿元。凤城八路立交等26项道路工程建成投用，开工建设公共停车位1.3万个、完成8523个。地铁5条在建线路加快推进，4号线实现轨通。运营线网日均客运量165.8万人次，客流强度居全国前三。地铁三期规划编制完成。西南郊水厂开工建设，湾子水厂全面竣工。新建改建110千伏变电站17座，增容109.4万千伏安。增加集中供热面积1274万平方米、天然气居民用户25万户。小寨地区海绵城市建设全面启动。开工干支线管廊38.4公里、缆线管廊86公里，昆明路综合管廊成为全国示范工程。实现手机扫码乘地铁、坐公交、办证照、交费用，市民生活更加便利。

（八）弥补民生短板增福祉。全市财政用于民生的支出达到837.2亿元，占一般公共预算支出的80.1%。开展就业创业“九个一”系列活动[21]，城镇新增就业15.46万人，农村劳动力转移就业78.89万人，城镇登记失业率控制在3.3%的较低水平。

聚焦精准脱贫，“四梁八柱”政策体系逐步建立。497支工作队驻村帮、2.4万名干部结对帮、社会各界合力帮，把贫困群众当亲人，用心用情用力真扶贫。实施易地搬迁安置项目19个，落实产业扶贫项目1031个，开发公益专岗2671个，改造危房4460户，健康、教育、金融、兜底保障等各项扶贫措施有效落实，涉贫重点区县、省定贫困村基本实现户户通电通水通路，民情地图、“三变”改革[22]、“十送”活动[23]等创新做法成为全省亮点。一年来，通过方方面面的努力，取得了实实在在的成效，贫困群众真真切切感受到了生活的新变化！

着力增加优质教育资源供给，大学区管理制改革深入推进，组建“名校+”教育联合体40个。开工建设公办中小学、幼儿园36所，完成义务教育学校“全面改薄”199所，增加学位3.8万个。完成农村学校旱厕改造，为50所中小学安装新风系统，建成梁家滩国际学校。评选表彰首批“最美教师”“最美少年”。20.13万外来务工人员随迁子女享受平等教育。民办教育得到进一步发展。

全面推行公立医院改革，建成各类医联体37个，改造提升乡镇卫生院100个、村卫生室2917个，农村医疗服务机构标准化建设全部达标。公立医疗机构取消药品加成，为群众减负3.66亿元。分级诊疗制度深入推进，组建家庭医生服务团队1955个。新农合参合率达99%，补偿参合患者1193万人次、总额达23.5亿元。落实鼓励社会办医各项政策，批准设置民办医疗机构237所。群众看病难、看病贵问题得到缓解。

推动社会保障全面提标，城乡低保标准分别提高到每人每月640元和400元，企业退休人员基本养老金实现“十三连涨”。城乡居民养老和医疗保险参保率分别达到99.7%和97.1%。建成保障性住房6904套，为新毕业大学生提供公租房1.91万套，累计分配公租房11.8万套。新增住房公积金贷款134亿元，使53.6万户家庭改善了住房条件。新建城市社区居家养老服务站112个、农村幸福院216个，新增养老床位6544张。不断完善“1+N”新型社会救助体系[24]，困难群众生活有了更好保障。

大力发展公共文化事业，“音乐之城”“书香之城”全面推进，戏剧惠民演出1559场，农村公益电影放映35000余场。陕西大剧院建成投用，农村文化礼堂建设试点全面展开。举办首届西安国际马拉松赛、第3届中国西安国际少儿美术节、全国青少年足球冠军杯赛、第16届西安市运动会等大型文体活动。人民群众的精神文化生活不断丰富，践行社会主义核心价值观的风尚更加浓郁，涌现出了李国武、黄忠文、吴一帆等一大批感动西安的身边好人。

深入推进平安西安建设。创建省级食品安全示范区（县）5个、餐饮服务示范街区43条，食品药品抽检合格率均在95%以上。刑事接报警数同比下降12.7%，社会治安满意度同比提高8.6个百分点。突发事件发生率下降11.6%，未发生重特大安全生产事故。公众安全感满意度达到93.5%。

（九）强化政府自身建设转作风。牢固树立“四个意识”，深入学习宣传贯彻党的十九大精神，忠实践行习近平新时代中国特色社会主义思想的意识更加坚定自觉。主动接受市人大及其常委会法律监督和工作监督，自觉接受市政协民主监督，办结建议、提案1155件，办复率100%。政府常务会学法常态化，市政府及工作部门实现法律顾问全覆盖。落实“三重一大”集体决策制度[25]。聘请市政府经济、科技、文化、金融等顾问82名，成立国际专家顾问团，加强与高校、社会智库等机构的合作，不断强化科学决策的智力支撑。强力推进“行政效能革命”，公布“最多跑一次”事项13675个，取消下放调整70项行政事权，清理规范12项行政审批中介服务事项，政府“瘦身健体”激发了强大的市场活力。“12345”市民热线综合服务平台建成投用，各级政务服务机构为企业和群众办事1334万余件，人民网网民留言办理连续7年获全国先进。严格廉洁从政，坚决彻底肃清魏民洲等流毒影响，党风廉政建设和反腐败斗争深入推进。

民族、宗教、参事、双拥、人防、统计、档案、侨务、地方志、气象、防震减灾、妇女儿童、残疾人等工作取得新进步。

各位代表！刚刚过去的2017年，是西安发展历程中极不平凡的一年。一年来，我们不忘初心，牢记使命，砥砺奋进，聚焦“五个扎实”、奋力追赶超越的思想更加统一、行动更加自觉；我们抢抓机遇，把握关键，精准发力，落实“五新”战略任务、推动大西安大发展的思路更加明晰、措施更加有力；我们紧盯短板，创新举措，全力攻坚，破解发展难题、推动“建强创佳”的方法更加科学、效果更加明显；我们不畏艰险，淬火历练，勇于担当，锻造西安铁军、真心干事创业的氛围更加浓厚、士气更加高涨。这一年，我市各级干部都很拼！我要为我们的干部点赞！

特别是在“聚焦三六九，振兴大西安”宏伟蓝图的鼓舞激励下，全市上下汇聚起追赶超越发展最广泛的思想共识和磅礴的奋斗力量，大家撸起袖子加油干，一天当做两天干，干成了一大批打基础、利长远、惠民生的实事要事，取得了前所未有的工作成效。西安在全省的“龙头”作用显著增强，在全国的战略地位显著提高，在全球的影响力和吸引力显著跃升。这一年，西安很出彩！我们要为全市人民点赞！

成绩来之不易，需要倍加珍惜。在此，我代表市政府，向全市广大干部群众，向各位代表、各位委员、各民主党派、人民团体和社会各界人士，致以崇高敬意！向所有关心支持西安发展的中省单位、广大企业，解放军、武警部队官兵和政法干警，以及海内外同胞、侨胞、友人，表示衷心感谢！

在总结成绩的同时，我们也清醒地认识到：我市发展不平衡不充分问题比较突出，拥有的资源禀赋和比较优势尚未充分发挥，补短板任务依然艰巨；市场化程度不够高，要素配置不尽合理，质量提升和动能转换还需持续用力；生态环境保护、治污减霾任重道远，教育、医疗、交通、养老等方面与人民日益增长的美好生活需要还有较大差距；政府职能转变仍需深化，依法行政能力、政务服务水平、应急机制建设等还需进一步提升。对这些问题，我们要结合加快补齐“十大短板”[26]，认真加以解决。

二、2018年工作总体要求和主要目标

2018年是贯彻党的十九大精神的开局之年，是改革开放40周年，是决胜全面建成小康社会、实施“十三五”规划承上启下的关键一年。最近，国务院正式批复了《关中平原城市群发展规划》，明确提出西安建设国家中心城市的发展定位，进一步标定了西安以深度融入“一带一路”建设为统领，打造具有历史文化特色和亚欧合作交流的国际化大都市的战略目标，使全市追赶超越的方向更加明确、路径更加清晰、信心更加坚定。我们一定要抓住这一难得的历史机遇，积极开展“追赶超越奋进年”活动，坚持低调务实不张扬、埋头苦干，以时不我待、只争朝夕的状态投入工作、奋力拼搏，努力开创新时代大西安大发展的新局面。

今年政府工作的总体要求是：高举习近平新时代中国特色社会主义思想伟大旗帜，认真贯彻党的十九大精神，按照中央和省市委部署要求，坚持新发展理念，坚持以人民为中心的发展思想，坚持稳中求进工作总基调，按照高质量发展要求，以供给侧结构性改革为主线，紧扣“追赶超越”和“五个扎实”，围绕“五新”战略任务和“聚焦三六九，振兴大西安”目标，紧盯国家定位、抢抓战略机遇、聚力改革创新、全力释放优势、着力补齐短板、奋力追赶超越，统筹抓好稳增长、促改革、调结构、惠民生、防风险各项工作，打好防范化解重大风险、精准脱贫、污染防治的攻坚战，促进经济社会持续健康发展，推动大西安、国家中心城市和亚欧合作交流的国际化大都市建设迈出坚实步伐。

综合考虑各方面因素，今年全市主要预期目标为：生产总值增长8%左右，规模以上工业增加值增长7.5%左右，全社会固定资产投资增长7%左右，社会消费品零售总额增长9%左右，一般公共预算收入增长8%以上，城乡居民人均可支配收入分别增长7%和8%左右，城镇登记失业率控制在4%以内，居民消费价格指数涨幅控制在3%左右，万元生产总值能耗下降3%，主要污染物排放总量削减完成省上下达任务。这一系列指标，既贯彻了中央和省上的总体要求、又切合我市的发展实际，既考虑了速度和质量、又兼顾了需要和可能，既反映了追赶超越的要求、又体现了做好“龙头”的担当。这些目标，都不是轻轻松松就能完成的，需要我们扎扎实实、创造性地开展工作，用最大的努力争取最好的结果。

做好今年政府工作，需要把握好以下几个方面：

——必须坚持以习近平新时代中国特色社会主义思想和党的十九大精神为指引。按照抓首要、大学习、促发展的要求，着力在学懂弄通做实上下功夫。牢固树立“四个意识”，不断增强“四个自信”，切实提高政治站位，自觉把思想和行动统一到党中央对形势的科学判断和各项决策部署上来，把“八个明确”“十四个坚持”[27]贯穿到我市统筹推进“五位一体”总体布局和协调推进“四个全面”战略布局的各个方面和全过程，始终以这一重大思想引领大西安追赶超越发展，加快迈向现代化、国际化。

——必须坚持推动高质量发展与做大经济总量相同步。认真贯彻习近平新时代中国特色社会主义经济思想，围绕加速迈入“万亿俱乐部”目标，以高质量发展统筹高端产业和高速增长，充分发挥投资、消费、出口“三驾马车”和改革、科技、文化“三大动力”协调拉动作用，在做大总量的同时做优质量，在做优质量的前提下做大总量。以供给侧结构性改革为主线，推动经济发展质量变革、效率变革、动力变革，着力提高全要素生产率，着力激发各类市场主体活力，着力打造“3+1”万亿级大产业[28]，加快构建具有西安特色的现代化经济体系，不断增强我市经济创新力、竞争力和辐射力。

——必须坚持稳中求进与追赶超越相统一。稳中求进是基调，追赶超越是定位。要切实把握好“稳”和“进”的关系，确保经济发展的基本面持续稳中向好，防止出现大的波动，同时在重点领域和优势强项上要有所进取、有所突破。要切实把握好“赶”和“超”的节奏，既要坚定信心、全力追赶，又要结合实际、遵循规律；既要全面对标、缩短差距，又要立足优势、勇于超越；既要“补短”增加“总分”，又要“扬长”争取“加分”。在稳中求进的前提下，确定好方向、选择好路径、掌握好平衡，努力实现高质量、高效益的追赶超越。

——必须坚持大西安、国家中心城市、亚欧合作交流的国际化大都市建设相衔接。当前和今后较长一段时期，是大西安建设、国家中心城市建设、亚欧合作交流的国际化大都市建设的历史交汇期、黄金机遇期、任务叠加期。三者之间，既是辐射带动的圈层展开，也是发展目标的梯次推进，必须科学谋划、有效实施。要尊重城市发展规律，按照“五个统筹”要求[29]，精准把握建设的有序推进、发展的融合衔接和工作的力度节奏，全力推进西咸一体化、富阎一体化，加快构建大西安都市圈；建好“三中心两高地一枢纽”，不断完善国家中心城市功能；打造“五都一枢纽”[30]，全面加快国际化进程，积极实施大西安新时代“三步走”战略[31]。

——必须坚持抓重点、补短板、强弱项相协调。强化系统思维，掌握科学方法，学会“十个指头弹钢琴”。既要统筹推进、也要抓住重点，既要厚植优势、也要补齐短板，既要巩固强项、也要提升弱项。要在依托突出区位交通优势，大力发展枢纽经济、门户经济、流动经济方面聚焦发力；在弥补工业短板，打造新能源汽车、新一代信息技术等“拳头”产业方面勇于突破；在破解科教、文化资源潜力释放不足，促进科创、文旅产业大发展方面争创亮点。找准工作的切入点和着力点，“一锤接着一锤敲”，以重点突破带动整体推进，用补短强弱促进协调发展，推动全市经济高质量发展，努力当好全省追赶超越的“排头兵”。

三、2018年重点工作任务

（一）推动高质量发展，着力构建具有西安特色的现代化经济体系。

持续深化供给侧结构性改革。落实我市“1+6”行动计划[32]，深入推进“三去一降一补”。大力破除无效供给，对僵尸企业实行清单管理。建立多主体供给、多渠道保障、租购并举的住房制度，确保商品房库存去化周期稳定在合理范围。强化政府债务管控，坚决守住不发生系统性金融风险底线。落实降成本行动计划，年内降低企业成本90亿元以上。加快实施我市“10+N”系列补短板行动方案[33]，不断增强发展的协调性、均衡性和可持续性。深化土地供给侧结构性改革，开展新一轮土地利用总体规划编制试点，以土地利用方式转变促进城市发展方式转型。

推动产业发展加快迈向“双高端”。围绕建设西部经济中心，不断提升产业发展的规模层次和质量效益，使我市产业发展既有“众木成林”、又有“参天大树”。

——大力发展战略性新兴产业和先进制造业，加快培育“拳头”产业。依托华为、中兴、阿里巴巴、腾讯、三星、美光、应用材料等重点企业，加快发展集成电路、智能终端、大数据与云计算、物联网和第5代移动通信技术，打造千亿级全球新一代信息技术产业基地。依托陕汽、比亚迪、法士特、吉利、开沃等骨干企业，围绕提升整车设计、三电（电驱、电池、电控）研发、系统集成及轻量化研究等核心技术，在扩大整车产能的同时，加快汽车零部件配套产业发展，打造百万辆全国新能源汽车制造重镇。依托航空、航天基地以及中航西飞集团、中航一飞院、中航试飞院、航天五院西安分院、航天六院等企业和科研院所，做好ARJ21和MA700支线客机、C919大型客机零部件、无人机以及北斗导航、工业机器人的研发生产，打造具有国际影响力的航空航天产业高地。持续壮大电力装备、新材料、新能源、医药制造等产业集群。积极创建“中国制造2025”国家级示范区，推进“两化”深度融合[34]，实施“三换三名”工程[35]，抓好100个市级重点技改项目，促进传统优势产业转型升级。

——加快服务业高端化发展。突出抓好创意设计、科技研发、检验检测、商务咨询等高端环节，完善法律、会计、信息与数据等服务体系建设，推动生产性服务业向专业化和价值链高端延伸。围绕打造时尚消费中心，抓好钟楼、西咸、浐灞等国际化商业中心建设，推进小寨、高新、曲江、经开等商圈提档升级，加快宜家荟聚购物中心等一批大型商业综合体建设，新增5个市级以上特色商业街，推动生活性服务业向精细化和高品质转变。加快国家电子商务示范城市建设，积极实施“电子商务倍增计划”，促进实体零售转型发展和线上线下融合发展，力争电子商务交易额达到3600亿元。全年举办规模以上会展活动200场以上，综合收益达到130亿元。

——全面提升金融服务水平。围绕打造丝路国际金融中心，加快建设科技、文化、军民融合3个金融示范区和能源、物流、绿色3个金融功能区，积极培育互联网金融、消费金融、融资租赁、商业保理等新金融，鼓励发展创业投资、私募股权投资、产业投资等基金，吸引更多境内外金融机构和高层次金融人才向西安聚集。实施“龙门行动计划”，新增境内外上市挂牌企业30家，实现融资300亿元。全年金融业占生产总值比重稳定在10%以上。

强力打造特色小镇“西安名片”。落实好中省特色小镇建设系列政策和我市《创建导则》，坚持产业“特而强”、功能“聚而合”、形态“小而美”、机制“新而活”，突出生产、生活、生态“三生融合”，重点围绕硬科技、文化旅游、军民融合等优势资源，加快建设50个左右特色小镇，体现西安特色，形成“西安模式”。

实施“新经济新业态壮大计划”。推动物联网、虚拟现实、增强现实等新技术与实体经济深度融合。积极发展众创、众包、众扶、众筹等新模式，支持人工智能、增材制造、大数据等新产业聚集发展，加快发展数字、共享、智慧、绿色经济等新业态，在中高端消费、现代供应链、人力资本服务等领域培育新的增长点。

大力振兴实体经济。实施“市场主体升级计划”和“科技小巨人企业培育计划”，加快培育一批营业收入过百亿的大企业大集团和行业龙头企业，发展一批“专精特新”中小企业，扶持小微企业灵活发展，全年净增“五上”企业500户以上，新增小巨人企业260家、各类市场主体20万户。深入推进质量强市战略，加快实施“标准化+”和“质量提升”行动，大力弘扬“工匠精神”，引导企业增品种、提品质、创品牌。

实施“民营经济倍增计划”。落实我市《关于促进民营经济加快发展的若干意见》，着力破解融资难题，搭建信息交流平台，构建“亲”“清”新型政商关系，进一步提振民企发展信心，民间投资增长6%以上。大力弘扬优秀企业家精神和新时代西商精神，积极营造“企业家功高、纳税者光荣”的社会氛围，努力打造关心重视企业家和企业家创新创业的双示范城市。

（二）深入实施创新驱动战略，着力培育壮大发展新动能。

全力打造全球硬科技之都。落实我市发展硬科技产业“十条措施”，统筹设立1000亿元产业引导基金。结合硬科技“八路军” [36]的发展需求，争取国家科技创新“2030重大项目”，吸引更多国内外知名科技组织和企业落户西安。加快推进中科院西安科学城建设，启动实施光启、汉能、大疆等一批硬科技、黑科技项目。协力打造西安交大西部创新港、西北工大翱翔小镇、西电科大军民融合创新谷、中兴科创小镇等11个具有国际水准的硬科技小镇。全力办好2018全球硬科技创新大会，持续扩大西安硬科技品牌的世界影响力。

加快推进“两区”建设。聚焦统筹科技资源、深化军民融合两大改革任务，坚持复制推广改革经验与深化提升创新成果同步推进。拓展科技大市场功能，推广“一院一所一校”改革经验[37]，全市技术合同交易额达到850亿元，就地转化率超过30%，研发投入占生产总值比重保持在5%以上。扎实推进国家知识产权强市和运营试点城市建设，支持建好国家知识产权军民融合运营平台和中国（西安）高端装备制造产业保护中心。推动军工企业混合所有制改革和军工科研院所事转企改革，统筹抓好军民融合“两园三基地”[38]建设，积极创建“国家军民融合标准化试点城市”。全年军民融合产业营业收入达到2500亿元以上，民参军企业数达到430家以上。支持高新区自创、自贸“双自联动”发展，打造引领创新发展、支撑开放合作的“双示范”样板区。

大力推进“双创”工作。完善“5552”成长格局[39]，加快建设各类众创载体。支持西安交大、西北工大、西电科大、光机所等高校院所发挥资源优势，建设高水平、专业化众创载体和创新创业学院，助力阿里巴巴、亚马逊、猪八戒、蒜泥科技等众创载体做大做强，全市众创空间达到450家以上、总面积1600万平方米以上。扎实推进国家小微企业创业创新基地城市示范，加强“双创”与产业的有机融合及对实体经济的有力支撑，大力发展“大学生创业经济”“校友经济”“院士经济”“院所经济”“教师经济”等五大创新创业经济。推行户籍新政2.0版[40]，提供人才安居房不少于5万套，人才安居货币化补贴实现全覆盖。办好2018西安国际创业节、西安国际创业大赛和“创业西安行”等活动，持续开展“百万大学生留西安就业创业”和“梦回长安——百万校友回归”活动。“种好梧桐树，引得凤凰来”。让世界各地的创新创业资源向西安加速奔流，让富集涌动的创新创业活力在西安加速释放，着力打造“一带一路”创新创业之都。

稳步推进重点领域改革。不断深化“放管服”改革，继续抓好商事制度改革，全面实施市场准入负面清单制度，加快推进企业简易注销登记和全程电子化，基本实现群众和企业办事“最多跑一次”。支持莲湖、高新、西咸等开展相对集中行政许可权改革试点，做实“一枚图章管审批”。完成公共资源交易平台整合，公共资源交易中心挂牌运营。完善代管西咸新区的体制机制，新区事新区办。深入推进国资国企改革，通过增资扩股、改制上市、并购重组以及分离移交社会职能等方式，促进国资增效益、国企增活力。推动国税、地税征管体制改革，深化财政专项资金管理改革。开展“营商环境提升年”活动，落实省上“十大行动”方案[41]，建立营商环境考核指标体系，完善“1+12+18”政策体系[42]，狠抓政策落地“最后一公里”，深入整治规范市场秩序，打造审批事项最少、办事效率最高、投资环境最优的政务生态体系，真正使西安成为企业成长的“乐土”、创业者圆梦的“福地”。

（三）主动融入“一带一路”大格局，着力打造国际重要门户枢纽。

大力发展枢纽经济、门户经济、流动经济。加快建设国家综合交通枢纽，推动空港、陆港、高铁港“三港联动”，不断提升西安的枢纽地位，增强在全国乃至全球的资源配置能力。启动西安咸阳国际机场三期扩建，新开通10条国际客运航线和4条国际货运航线，力争机场客运量超过4500万人次、货运量达到30万吨，打造国家临空经济示范区。充分发挥“米字型”高铁网络突出优势，加快建设高铁新城。启动北客站交通综合枢纽中心建设，年内北客站至机场城际铁路建成通车，打造区域“换乘中心”。加快建设“2环+12辐射”[43]对外高速公路大通道，全面提升大西安交通通达能力。加强与沿海、沿边口岸通关协作，加快构建大通关综合服务体系，充分发挥向西开放、向东集散、辐射全国的门户作用，打造多元化开放“新门户”。不断完善综合保税区功能，支持空港保税物流中心升级为综合保税区，加快国际贸易“单一窗口”推广应用。拓展“长安号”中欧班列业务范围，加密运行频次，力争年内开行1000列。积极构建现代化、智慧化物流体系，推动城市共同配送，促进人流、物流、资金流、信息流加速流动，打造区域“集散转运中心”。扎实做好国家现代物流创新发展和供应链体系建设“双试点”工作，推进西安港冷链物流仓储基地、新筑铁路综合物流中心、空港新城航空物流枢纽三大聚集区建设，加快实施海航、京东、传化、菜鸟等重大物流项目。积极办好第8届中国西部物流博览会。全年物流业增加值增长15%以上。

高水平建设自贸试验区。坚持以制度创新为核心，加快建设与国际投资贸易规则相适应的政务服务体系。全面落实127项改革试点任务，促进资本集聚、产业集聚，入区企业达到1万家以上。加快跨境人民币结算中心建设，提升金融改革创新和便利化服务水平。建立“一带一路”互联互通合作机制，探索建设内陆型自由贸易港。让自贸试验区成为打造内陆型改革开放新高地的先导区，使西安由“跟跑”开放向“引领”开放转变。

积极做好对外经贸合作。加快建设欧亚经济综合园区核心区、中俄丝路创新园、中欧合作产业园等国际产能合作项目，支持建立“一带一路”产业园区发展联盟。坚持优进优出，持续加大先进技术设备和关键零部件进口，不断扩大高新技术和机电产品出口，着力打造一批具有国际竞争力的服务出口品牌。加快发展跨境电子商务。全年进出口总值达到2800亿元。积极拓展“海外西安”新空间，发挥好陕鼓、西电、陕汽、爱菊、隆基股份、大唐西市等“先遣军”的示范带动作用，鼓励支持更多本土优质企业“走出去”，在参与全球化竞争中提升西安企业的品牌影响力。

提升城市公共服务国际化水平。加快西安领事馆区建设，争取哈萨克斯坦总领事馆、尼日利亚商务中心年内启用。发展更多国际友好城市，策划建设友城街区，推动友城之间互设城市展示、产品展销平台。加快建设国际社区、学校、医院等国际化公共服务设施，积极引进国际商协会、国际中介服务机构，进一步完善国际标志标识，促进公共服务系统多语化建设，提升市民国际化素养，让所有在西安的外籍人士都能感受到方便、体会到融洽、触摸到这座城市的温度，真正爱上西安、更好融入西安。

（四）坚定文化自信，着力建设世界文化之都。

实施中华优秀传统文化传承发展工程。持续加大对明城墙、汉长安城、秦咸阳宫等各类遗址和文物的保护展示力度，加快推进碑林、小雁塔、半坡遗址等历史文化片区改造提升，推动建设杜陵国家遗址公园，坚决守护好人类文明遗产和民族精神家园。继续抓好博物馆之城建设，年内新增5座以上博物馆。传承红色文化基因，实施革命旧址保护和教育展示工程，抓好七贤庄红色文化示范街区建设。加强非物质文化遗产保护。实施中华老字号保护发展工程。深入挖掘古都历史文化价值，运用现代科技手段推动中华优秀传统文化融入新时代，培育和践行社会主义核心价值观，进一步凝聚城市精神，强化文化担当，用西安的方式讲好中国故事，用文化的力量展示中国风采。

推动文化旅游产业升级跨越。实施文化、旅游两个产业倍增计划，推进“文化+”“旅游+”，打造万亿级文化旅游大产业。加快建设以曲江新区为引领的“文创大走廊”，促进“十大文化产业园区”[44]加速发展，培育一批实力雄厚、竞争力强的“文化航母”企业，确保文化产业增加值增长15%以上。围绕“文化+人脑+电脑”，大力发展文化创意、数字出版、动漫游戏、电子竞技、文化装备制造等产业，支持丝路国际创意“梦工场”、明德门文化艺术创意小镇、丝路文旅小镇等加快建设。持续推进“名城、名家、名作”工程，做强做精做优西安影视、仿唐乐舞、西安鼓乐、秦腔等特色文化品牌，加快推出一批文化精品力作，用文化艺术的形式演绎新时代大西安的精彩崛起，为提升中国文化的世界影响力贡献“西安力量”。

围绕打造世界旅游时尚之都，积极实施“旅游国际化行动”，着力抓好国家级旅游业改革创新先行区和全域旅游示范市创建工作，推动旅游与文化、科技、会展、商贸、体育等深度融合，全面发展优质旅游。持续抓好城墙·碑林、朱雀·太平、翠华山·南五台和大明宫4个5A级景区创建工作，加快华侨城系列文旅、宋城集团“中华千古情”、华夏文旅综合体二期等文化旅游项目建设。构筑月光经济“一极两轴五板块多节点”[45]发展格局，开展城墙景区灯光秀、快乐跑等主题活动，打造“夜游西安”品牌。开展“西安年·最中国”系列活动，丰富西安旅游内涵。用好“国际美食之都”金字招牌，大力发展美食经济，打造“舌尖上的西安”。完善旅游软硬件设施配套，实施旅游厕所革命“三年行动计划”，加快发展“智慧旅游”、打造智慧景区，规划建设一批高星级酒店群，深入整治规范旅游市场秩序，全面提升旅游服务质量。策划西安城市形象设计，加大西安国际品牌营销。全年接待游客数量超过2亿人次、旅游业总收入超过2000亿元。

加强国际人文交流合作。围绕建设对外交往中心，搭建国际人文交流平台。积极申办、筹备2019“一带一路”国际合作高峰论坛，加快建设西安丝路国际会展中心。全力办好第3届中法文化论坛、中德历史文化名城对话会、丝绸之路国际艺术节和电影节、全球无人机大赛等国际交流活动。支持高校提升国际化办学水平，推动国际学术交流。鼓励更多本土文化企业、团队、人才“走出去”，传播“西安好声音”，讲述“古城新故事”。

推进现代公共文化服务体系建设。健全四级公共文化设施网络，构建大西安“文化通”数字移动平台，促进文化共建共享。启动西安图书馆新馆等一批公共文化设施建设，新建一批社区书屋和书香西安阅读吧，组织开展“万人万卷·阅动西安”全民阅读、丝绸之路国际图书博览会等系列活动，聚力打造“书香之城”。继续推进农村文化礼堂建设，全年放映公益电影37000场，文化惠民演出1500场。扎实做好第14届全运会各项准备工作，加快西安奥体中心等场馆建设，办好2018西安国际马拉松赛等体育赛事，广泛开展群众性体育健身活动，增强市民健康体魄，展示城市运动之美。

（五）聚焦人居环境宜居化，着力建设品质西安。

优化城市空间布局。围绕“三轴三带三廊一通道多中心多组团”[46]发展格局，积极实施“北跨、南控、西进、东拓、中优”战略，推动大西安从“围城”建设时代进入“拥河”发展时代。开展“品质规划攻坚年”活动，启动新一轮城市总体规划修编工作，推进多规合一。加强中心城区规划管控，坚持“只拆不建、多拆少建”，疏解建筑、人口密度。系统推进“城市设计”和“城市双修”[47]试点，塑造特色风貌，推动有机更新。支持西咸新区加快建设大西安新轴线、新中心。主动加强与咸阳、渭南的对接融合，启动富阎产业合作园区首期10平方公里建设，加快西咸、富阎一体化进程。

不断完善城市功能。落实《品质西安建设补短板三年行动方案》，持续推进“强基增容”工程，全年完成城建投资710亿元。加快地铁在建项目建设，4号线年底前建成通车。抓紧报批第三期地铁规划并组织实施。进一步提升地铁运营水平，优化公交线网布局，公共交通出行分担率达到60%以上。抓好昆明路、西延路、建工路等快速路建设，加快西铜一级公路（西安—高陵）、经九路、酒十路等市政道路改造，启动西三环—阿房一路等立交工程。打通25条断头路，开工建设32个公交场站和1.5万个停车位。加快实施智能电网、户表集中改造、老化供水供热管网改造工程。建成投用第二气源，启动建设气化三期。实施城市地下空间规划建设利用“三年行动”，抓好年度20个示范项目。开工干支线管廊40公里、缆线管廊90公里。加快高新、曲江、浐灞、常宁新区等海绵城市试点，小寨地区海绵城市建设初具规模。继续推进火车站、幸福路等城市片区改造，提升区域功能和城市品质。

持续抓好“四治一增”。坚持铁腕治霾，按照“治霾十法”[48]，加大产业结构和能源结构调整力度，打好“减煤、控车、抑尘、治源、禁燃、增绿”组合拳。加快推进清洁供暖、燃气锅炉低氮改造和农村煤改气（电）等工作，强化重点排放企业全程监管。建成“智慧环保综合指挥中心”，实现线上线下同步监管，不断提升网格化管理实效。加大关中城市群联防联控力度，合力打赢蓝天保卫战，全年优良天数不少于200天。抓实抓好“河长制”“湖长制”，坚持“五策治水”，深化“8+5+2”河湖水系治理[49]，实施“剿劣水”行动，推进渭河断面水质达标、渼陂湖等8大重点水利工程[50]建设，新增生态水面2300亩、湿地5000亩，加快恢复“八水绕长安”生态美景。依法保护秦岭生态环境，强化“四治一专两核查”[51]常态化监管，建立多元化生态补偿机制，继续实施环山公路“三化”工程及特色节点建设，坚决守好国家生态安全屏障，不断增强“城市绿肺”功能。深入推进“烟头革命”，持续提升保洁水平。实施城区厕所革命“三年行动计划”，新建公厕440座、改造提升666座、鼓励开放380座以上。强力推进“四改两拆”，启动城棚改项目16个，改造老旧小区200万平方米、旧厂区项目14个，实施架空线缆落地193公里，整治拆除违法建设800万平方米以上，基本完成违法户外广告及牌匾标识拆除任务。实行生活垃圾处理区域补偿机制，加快5个生活垃圾无害化处理项目建设，年内建成八兴滩餐厨垃圾无害化处理厂。持续开展“五路”两侧增绿美化、“两路两侧三化”[52]整治和“美丽西安·绿色家园”行动，新建公园4个、绿地广场60个，新增城市绿地面积500万平方米、立体绿化面积10万平方米，植树造林3万亩，打造一路一景、三季有花、四季常绿的绿色之城、花园之城。支持西咸新区、浐灞生态区加快建设国家生态文明先行示范区，探索形成可复制、可推广的生态文明建设模式。

加快建设智慧西安。建成城市运行大数据中心和政务服务平台，实现信息资源开放共享。积极拓展智慧交通、智慧医疗、智慧校园、智慧社区等服务领域，加快推动面向市民的公共服务信息化建设，推进更多领域实现移动支付、手机办事，建设“指尖上的城市”。年内实现主城区刷手机乘公交全覆盖。落实“城市管理要像绣花一样精细”的要求，全面提升城市精细化管理水平，大力开展“互联网+网格化”行动，加快实施“雪亮工程”[53]，让城市插上智慧的翅膀。

（六）积极实施乡村振兴战略，着力推进农业农村现代化。

坚决打好精准脱贫攻坚战。聚焦“12345”目标[54]，注重扶贫同扶志、扶智相结合，下足绣花功夫，做到脱真贫、真脱贫。持续加大财政投入，市级财政专项扶贫资金较上年增加20%。实施“十百千万”产业扶贫工程[55]，开展就业扶贫行动，确保有正常劳动能力的贫困家庭每户有1人稳定就业，涉贫区县年内至少建成1个返乡创业园区。全面完成危房改造和移民搬迁，高标准落实教育、健康、兜底保障政策，贫困村基础设施和公共服务全面达标。积极对接落实苏陕扶贫协作项目，确保周至县脱贫摘帽。年底全市142个贫困村、12864个贫困户、40570名贫困人口（不含兜底保障人员）达标退出，在全省率先实现全面脱贫，让贫困村的面貌焕然一新，让每一户贫困群众都衣食无忧、都有靠奋斗脱贫致富的志气！

加快发展都市型现代农业。深入推进农业供给侧结构性改革，大力发展苗木花卉、乡村旅游、休闲养生、特色民宿等产业，加快形成三产互动、跨界融合、良性循环的发展局面。推广蓝田“全域旅游”模式，开展田园综合体试点示范，全年休闲农业接待游客2500万人次，经营收入25亿元。推进电商“下乡进村”，培育一批集聚效应强的电商平台。加快灞源华润红色希望小镇和暖泉湾猕猴桃特色小镇建设，促进绿色农产品入网进场。优化“一区三带七板块”发展格局，新增市级以上现代农业园区15个、产业化龙头企业10家。大力发展绿色农产品，新增“三品一标”[56]农产品40个、无公害农产品基地40个。积极培育新型农业经营主体，年内培育家庭农场100家、职业农民1500人。全面落实粮食安全责任制，粮食产量达到180万吨以上。

扎实抓好农村环境整治。强化土壤污染管控和修复，加强农业面源污染防治，推进农村垃圾综合治理。实施农村厕所革命“三年行动计划”，年内改厕5万户。做好村容村貌绿化美化和村镇门户景观建设，凸显关中民居特色，年内创建5个美丽镇街、打造85个美丽村庄。着力建好、管好、护好、运营好农村公路，为农村聚人气、旺财气。

积极推进农村改革。深化农村集体产权制度改革，健全农村产权流转交易四级服务体系。总结推广蓝田董岭村“三变”改革经验，激活农村各类要素资源，拓宽农民增收渠道。完成国有林场改革任务。继续支持高陵区做好国家农村改革试点。深化农村金融改革，开展农户信用等级评价，打通金融服务“最后一米”，为“三农”引入金融活水。

实施“远郊区县域经济倍增计划”。充分发挥远郊区县空间优势，提升产业规划水平，积极承接城区产业转移。强化一区（县）一主业、一镇（街）一特色，一村一品牌，做精做专区县工业园、农业园，引导差异化发展，形成特色鲜明的区县域经济板块。按照“大干大支持”的原则，创新市财政支持区县发展的政策措施，加快补齐区县域经济短板，促进协调发展，实现富民强县。

（七）坚持项目带动战略，着力扩大有效投资。

加快推进项目建设。坚持“以市场换产业、以项目换投资”，紧盯新兴产业、基础设施、生态建设、社会民生等重点领域，储备一批事关全局和长远发展的重大项目。落实领导包抓市级重点项目制度，用好“互看互比互学”平台，做好水电气路等全要素保障，确保市级重点项目完成投资2900亿元以上。认真组织实施“招商项目落地年”活动，全力抓好已签约项目的跟踪落实，切实提高开工注册率、资金到位率和竣工投产率，为稳增长奠定坚实基础、提供有力支撑。

紧盯“五资”抓招商。更加注重招商引资的质量效益和实际效果，瞄准世界500强、国内500强，努力引进一批大项目、好项目。围绕“3+1”万亿级大产业和优势产业集群，以龙头企业为依托，开展一对一联系、点对点衔接，实施产业链招商和资本招商，鼓励以商招商。全面落实与央企的战略合作，促进央企资金、技术、品牌优势与西安资源优势双向融合。高水平办好第2届世界西商大会、全球程序员节等重大活动。进一步创新政府投融资模式，再推出一批重大PPP项目，新增推介项目2000亿元以上。确保全年实际引进内资2890亿元、实际利用外资58亿美元。

切实提高投资质量。充分发挥有效投资对优化供给结构的关键性作用，进一步完善考核办法，加大产业投资的考核权重，促进各类资本向硬科技产业、文化旅游产业和新经济、新业态等领域聚集。切实抓好115个重点工业投资项目，确保强生全球供应链生产基地（一期）等30个重大项目年内建成投产，工业投资增长5%以上，占固定资产投资比重达到15%以上。充分释放民间投资潜力，吸引更多民间资本投资我市基础设施、社会事业和特色优势产业，力争年内引入民间资本达到3000亿元。

（八）坚持民生优先导向，着力满足群众日益增长的美好生活需要。

聚焦破解民生“九难”，持续推进“十个一”民生工程，扎实办好为民“十大实事”[57]，使人民群众的获得感、幸福感、安全感更加充实、更有保障、更可持续。

切实提高就业质量和收入水平。落实更加积极的就业政策，努力实现更高质量和更充分的就业，全年城镇新增就业12万人，农村劳动力转移就业65万人，实现“零就业”家庭动态清零。实施高校毕业生“3+N”就业促进计划[58]，消除就业歧视，构建和谐劳动关系。多措并举解决农民工工资拖欠问题，确保农民工都能拿到辛苦钱。拓展居民劳动收入和财产性收入渠道，不断提高城乡居民收入。

优先发展教育事业。以解决中小学生课外负担重、“择校热”“大班额”以及婴幼儿照护、儿童早期教育等问题为重点，努力办好人民满意的教育。新建标准化中小学、幼儿园30所，实施“全面改薄”154所，增加学位4万个，普惠性幼儿园占比达到65%。以大学区建设为抓手，年内组建25个“名校+”教育联合体，促进优质教育资源共享。改革民办学校“小升初”政策，完善小学“弹性离校”制度，扩大校园新风系统试点。全面完成普通高中省级标准化建设，稳步实施新高考制度改革试点。推进市属高校产教融合，支持8所高校创建“双一流”。实施第二期特殊教育提升计划，推动职业教育、民族教育、成人教育、民办教育等健康协调发展。认真落实《中共中央国务院关于全面深化新时代教师队伍建设改革的意见》，持续抓好师德师风建设，全面实施名师名校长“百千万工程”[59]，用教育家校长和高素质教师打造西安教育品牌，撑起千百万家庭的未来。

持续推进健康西安建设。深化公立医院综合改革，健全完善分级诊疗制度，建成紧密型医联体13个。继续实施城市公立医院对口帮扶基层医疗卫生机构，推动优质医疗资源“双下沉、两提升”[60]，办好群众家门口的医院。加强全科医生队伍建设，启动新一轮改善医疗服务三年行动计划，做实家庭医生签约服务。做好重点传染病专病专防。推动社会办医提质发展。加快建设大西安区域医学检验中心、市人民医院和鄠邑等4所区县医院[61]，争取国家健康医疗大数据中心及产业园区项目尽快落地。深入推进全国养老服务业综合改革试点工作，鼓励支持社会力量参与养老事业，对2000户贫困老年人家庭进行适老化无障碍设施改造，建成医养机构20个、城市社区居家养老服务站35个、农村幸福院184个，新增养老床位6400张，不断造福全市老年人。

切实提升社会保障和公共服务水平。整合城乡居民医疗保险制度，建立城乡居民基础养老金正常调整机制，推进异地就医住院费用直接结算，加快社会保障卡发行应用，更好保障参保群众生活。适时提高城乡低保标准、特困供养人员保障标准和重度残疾人救助标准，加大社会扶助、救助力度，实现困难群众应保尽保、应助尽助。健全农村留守儿童和妇女、老年人关爱服务体系。有效增加公租房和限价房房源，年内新增租金补贴家庭2000户，继续加大房产办证遗留问题解决力度。建立完善物业服务企业诚信机制和监管机制，物业管理覆盖率提升至60%以上。完成37个农贸市场改造提升，规范各类早市和时令市场的管理，让市民享有更多获得感。

加快形成共建共治共享的社会治理格局。积极发挥社区、社会组织在社会治理中的作用，大力推广莲湖“律师进社区”经验，实现政府治理和社会调节、居民自治良性互动。组织实施好第10次村委会换届工作。推进“诚信西安”建设，逐步形成政府监管、公民自律、企业内控和社会监督“四位一体”的诚信体系。实施食品药品安全工程，让群众吃得安全、用得放心。加强质量技术监督，强化特种设备安全监管。严格落实安全生产责任制，加强城市安全风险分级管控，开展交通、消防、燃气、危险化学品等重点领域，以及人员密集场所、建筑工地、城中村、高层建筑等重点区域安全专项治理，坚决遏制重特大生产安全事故发生。按照中央“五个坚持”[62]要求，坚决打赢扫黑除恶专项斗争攻坚仗。加强治安防控体系建设，依法严厉打击暴力恐怖、电信诈骗、非法集资和传销等违法犯罪活动，打造“平安西安”。完善交通指挥体系，持续开展“车让人”行动，大力整治三轮车、摩托车非法载客行为，规范共享单车运营秩序。做好气象预警、防震减灾等工作，提升突发事件应急处置能力，筑牢城市安全“防火墙”，确保人民安居乐业、城市安全运行、社会安定有序。

扎实推进“七五普法”。做好优抚工作，深入开展双拥创建活动。切实做好第四次经济普查，进一步加强统计工作。继续做好民族、宗教、参事、人防、档案、地方志等工作。

四、全面建设人民满意政府

面对千帆竞发、百舸争流的城市间竞争，不进则退、慢进也是退。我们必须牢固树立以人民为中心的发展思想，准确把握社会主要矛盾变化的新特点，不忘初心、牢记使命、奋力拼搏，全面建设新时代人民满意政府。

一要充分发挥“头雁效应”。全市政府系统各级领导干部，要牢固树立“四个意识”，不断增强“八种本领”[63]，确保做到“五个过硬”[64]，推动中省各项决策部署在西安落地生根。充分发挥“关键少数”的示范引领作用，适应新形势、着眼新任务、植根新实践，用“真本事”“硬功夫”和“好口碑”，为干部群众立标杆、树榜样、做表率，真正形成一级做给一级看、一级带着一级干的良好工作氛围。

二要淬火锻造“西安铁军”。全市政府系统所有工作人员，都要积极践行“胸怀大局、无私奉献、弘扬传统、艰苦创业”的“西迁精神”，按照“西安铁军”标准和要求，驰而不息反“四风”、转作风、提效能、优服务，“甩开膀子下势干”“事不过夜马上办”。强化“以能力比高低、以实绩论英雄”的鲜明导向，让愿干事、能干事、敢担当的干部有舞台、有盼头、有底气，不断激发广大干部干事创业、比学赶超的内生动力，在实干中创造新业绩，在担当中彰显新形象，在奋斗中谱写新辉煌。

三要竭力当好“五星级店小二”。充分发挥“12345”市民热线、网上群众工作部和“千人亲商助企”等平台作用，持续推进“最多跑一次”改革，让政务上云端、服务接地气，让数据多跑路、群众少跑腿，真正使市民和企业办事既方便、又省心。要常进百家门、常解百家难，脚上多沾泥、争当好公仆，做到百姓之事多上心、百姓之忧多操心、百姓之盼多用心。

四要扎实推进廉洁政府建设。时刻把纪律和规矩挺在前面，严格落实中央“八项规定”精神和实施细则要求，始终保持惩治腐败“零容忍”高压态势，从严查处侵害群众利益的行为，既要干事、又要干净，坚决守好廉洁底线。构建系统完备、科学规范、运行有效的依法行政制度体系，做到“法无授权不可为、法定职责必须为”，切实把权力关进制度的“笼子”。自觉接受人大监督、政协监督，加强审计监督，主动接受群众和媒体监督，充分发挥《每日聚焦》《问政时刻》等栏目作用，始终把政府工作放在全社会的“聚光灯”下，着力打造“阳光政府”。

各位代表！西安有戏，合力才能好戏连台；人生有梦，奋斗才能梦想成真。目标已定，号角吹响，我们已经踏上新时代的“赶考路”。让我们更加紧密地团结在以习近平同志为核心的党中央周围，在市委的坚强领导下，凝心聚力、抢抓机遇，敢闯敢干、奋勇争先，为新时代大西安实现大发展、携手共创全市人民幸福美好的新生活而努力奋斗！
